# Advances in phage display based nano immunosensors for cholera toxin

**DOI:** 10.3389/fimmu.2023.1224397

**Published:** 2023-09-13

**Authors:** Yang Li, Kai-di Yang, De-cai Kong, Jun-feng Ye

**Affiliations:** ^1^ General Surgery Center, First Hospital of Jilin University, Changchun, Jilin, China; ^2^ School of Nursing, Jilin University, Changchun, China

**Keywords:** immunosensors, phage display, cholera toxin, diagnostic approach, nano biosensors

## Abstract

Cholera, a persistent global public health concern, continues to cause outbreaks in approximately 30 countries and territories this year. The imperative to safeguard water sources and food from Vibrio cholerae, the causative pathogen, remains urgent. The bacterium is mainly disseminated via ingestion of contaminated water or food. Despite the plate method’s gold standard status for detection, its time-consuming nature, taking several days to provide results, remains a challenge. The emergence of novel virulence serotypes raises public health concerns, potentially compromising existing detection methods. Hence, exploiting Vibrio cholerae toxin testing holds promise due to its inherent stability. Immunobiosensors, leveraging antibody specificity and sensitivity, present formidable tools for detecting diverse small molecules, encompassing drugs, hormones, toxins, and environmental pollutants. This review explores cholera toxin detection, highlighting phage display-based nano immunosensors’ potential. Engineered bacteriophages exhibit exceptional cholera toxin affinity, through specific antibody fragments or mimotopes, enabling precise quantification. This innovative approach promises to reshape cholera toxin detection, offering an alternative to animal-derived methods. Harnessing engineered bacteriophages aligns with ethical detection and emphasizes sensitivity and accuracy, a pivotal stride in the evolution of detection strategies. This review primarily introduces recent advancements in phage display-based nano immunosensors for cholera toxin, encompassing technical aspects, current challenges, and future prospects.

## Introduction

Cholera, a malady triggered by Vibrio cholerae, induces grave diarrhea and dehydration owing to its toxin production. Prevalent in areas with inadequate sanitation and restricted clean water access, this ailment poses a substantial threat to children under five, underscoring the significance of timely treatment to avert fatal consequences (1–3). Cholera’s profound influence on global health is evident in its annual toll of millions of cases and thousands of fatalities. Moreover, the economic ramifications are substantial, encompassing expenses for treatment, lost productivity, and public health interventions in affected nations (4–6). Cholera diagnosis entails laboratory testing, utilizing phage display-based nano immunosensors for cholera toxin detection. Early identification and treatment are vital for effective disease management. To combat cholera’s global impact, prevention measures like enhanced sanitation and clean water access are crucial. Vibrio cholerae, the causative bacterium, encompasses various strains, with serotypes O1 and O139 commonly associated with outbreaks. These strains produce a potent cholera toxin, inducing severe diarrhea and dehydration in infected individuals, hallmarking characteristic symptoms like watery diarrhea and vomiting.

**Figure 1:**
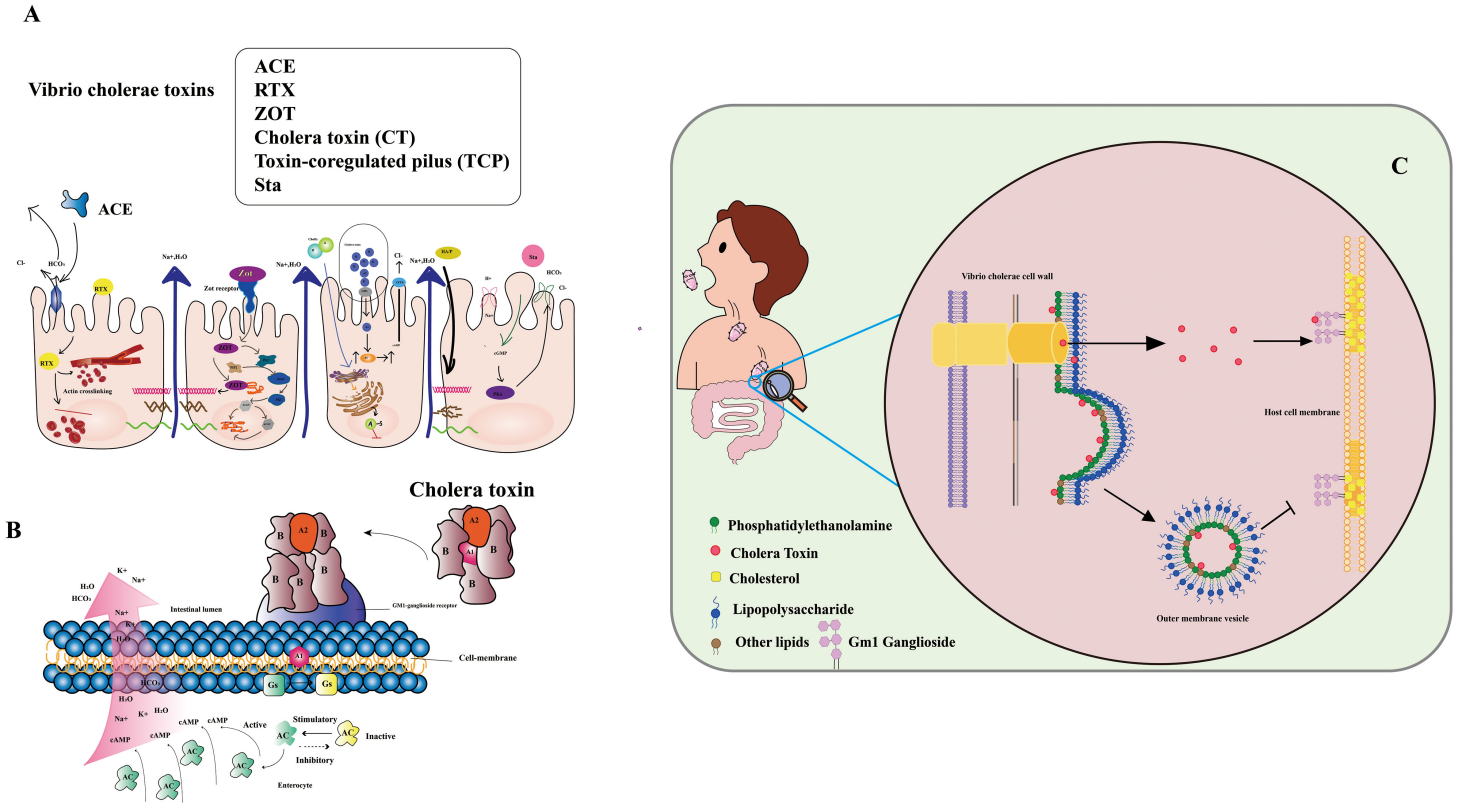
**(A)** Infection with Vibrio cholera. The Cholera Toxin (CT) binds to the Ganglioside GM1 and increases cAMP by increasing adenylate cyclase (AC) activity. Zonula occludens toxin (Zot) affects the structure of intestinal mucosal epithelial cells. Accessory cholera enterotoxin (ACE) stimulates Cl-/HCO_3_-secretion in enterocytes. Heat-stable enterotoxin (Sta) increases cGMP while inhibiting the Na+/Cl regulation. (Adapted from [8]) **(B)** Mechanism of action of V. cholera toxin. The complete toxin is shown to bind to the cell membrane’s GM1-ganglioside receptor. The A1 catalyzes the GS regulatory protein, “locking” it in active. Accumulation of cAMP along the cell membrane is caused by increased adenylate cyclase (AC) activity. (Adapted from [9]) **(C)** CT secretion via OMVs and through the T2S (type II secretion) system are complementary mechanisms for CT delivery, ensuring that the toxin can effectively penetrate and damage host cells (Adapted from [10]).

Vibrio cholerae secretes various toxins, intricately involved in cholera’s pathogenesis (7, 8) (**Figure 1A**). These encompass: 1. Cholera toxin (CT): A protein complex responsible for hallmark cholera symptoms - watery diarrhea and vomiting. The toxin binds to intestinal cell surfaces, triggering a signaling cascade that induces chloride and water secretion into the intestinal lumen. 2. Toxin-coregulated pilus (TCP): An indispensable surface protein for Vibrio cholerae colonization in the human intestine. Facilitating bacterial biofilm formation and adherence to intestinal cells. 3. Zonula occludens toxin (ZOT): A protein toxin disrupting tight junctions between intestinal cells, causing increased permeability of the intestinal epithelium and loss of water and electrolytes. 4. Repeat in toxin (RTX): RTX, a protein toxin, lyses host cells, liberating nutrients to fuel Vibrio cholerae growth and replication. 5. Accessory cholera enterotoxin (ACE): A protein toxin that potentiates cholera toxin activity, elevating water and electrolyte secretion into the intestinal lumen. 6. Cholerae-sensitive enterotoxin (Sta): A protein toxin intensifying cholera toxin activity and directly impacting the intestinal epithelium, heightening water and electrolyte secretion.

Vibrio cholerae primarily spreads via the fecal-oral route, stemming from direct contact with contaminated individuals or indirectly through fluids, food, or materials tainted by the pathogen (11). Widespread cholera outbreaks often correlate with natural or human-induced disasters that disrupt water and sanitation systems, facilitating disease transmission. The aquatic environment also acts as a key player in cholera spread, with the bacteria flourishing in waterways conducive to their proliferation (12, 13). The paramount importance of precise and dependable detection methods for monitoring and curtailing cholera transmission cannot be overstated (14). Thus, the imperative to devise effective, accurate, and reliable techniques for detecting pathogenic Vibrio cholerae in water sources becomes indispensable in mitigating the disease’s propagation (15, 16). Although the gold standard for cholera diagnosis remains isolating Vibrio cholerae O1 serotype from fecal samples, novel virulent serotypes demand alternative detection approaches (17). Given the stable nature of cholera toxin’s structure, detecting Vibrio cholerae toxin could offer significant advantages (18, 19). Cholera toxin (CT), a virulent protein produced by Vibrio cholerae, underpins disease symptoms. CT comprises CTA and CTB subunits, with CTA driving the disease phenotype and CTB serving as the vehicle transporting CTA to target cells. CTA consists of two major domains, CTA1 (the active toxin) and CTA2 (anchoring CTB). Conversely, CTB, a homotrimeric and non-toxic protein, exhibits robust binding to GM1 gangliosides on mammalian cells, displaying high affinity (20) (**Figure 1B**). CT lacks affinity for LPS, a major component of the outer membrane’s outer leaflet (OM). Nonetheless, certain CT molecules enter the periplasm and integrate into OMVs (outer membrane vesicles), small spherical structures budding from the OM. Unlike soluble CT, OMV-incorporated CT fails to bind to the GM1 receptor on host cell lipid rafts due to its location within the OMV. Thus, CT secretion via OMVs and the T2S (type II secretion) system synergize as complementary mechanisms for effective toxin delivery, enabling penetration and damage to host cells (**Figure 1C**).

State-of-the-art approaches for cholera toxin analysis, like high-performance liquid chromatography (HPLC) (21), liquid chromatography-mass spectrometry (22), gas chromatography (23), and thin-layer chromatography (TLC) (24), boast sensitivity and precision. However, their practicality for field testing or extensive screening can pose challenges. Nano immunosensors offer a promising avenue for toxin rapid identification, boasting simplicity, cost-effectiveness, high specificity, and field monitoring prowess. Several immunoassay techniques, encompassing enzyme-linked immunosorbent assay (ELISA) (25), fluorescence polarization immunoassays (26), fluorescent immunoassays (27), and side-flow immunochromatographic assays (IFA) (28), have been documented for cholera toxin detection. In these assays, antibodies, including monoclonal, polyclonal, and recombinant variants, serve as primary immune reagents. Competition-based formats are common due to the toxin’s small size, precluding simultaneous binding by both antibodies. A diverse range of antibodies targeting various cholera toxins, such as monoclonal antibodies (29), polyclonal antibodies (30), and recombinant antibodies (31), have been prepared. Currently, antibodies remain pivotal immune reagents and their preparation continues to evolve (32, 33). However, traditional antibody production can be intricate, time-consuming, and costly, hindering broad application (34). Specific antibody fragments or mimotopes present a compelling alternative for cholera toxin detection, as they exhibit precise binding to antibodies, competing with analytes for binding sites (35, 36). Mimotopes, peptides imitating protein, carbohydrate, or lipid epitopes, hold immense potential for diagnosis, immunotherapy, and vaccine development (37). With their vital roles in these fields, mimotopes emerge as a crucial asset (38–40). Nano immunosensors offer a promising departure from traditional immunoassays in toxin detection. By emulating toxin epitopes and mirroring their properties, nano immunosensors replace toxins or toxin-derived reagents in non-toxic analysis (41, 42). Notably, simulated peptides and anti-idiotype antibodies currently underpin toxin immunoassay development (43, 44). Incorporating a wide array of applications, phage display technology spans epitope mapping (45), enzymatic functions (46), targeted drug delivery (47, 48), and protein interaction definition (49, 50).

Recent research has centered on phage display-based nano immunosensors for cholera toxin detection. Utilizing phage-displayed peptides to mimic cholera toxin epitopes, these immunosensors enable rapid, sensitive, and specific detection in water samples. Nevertheless, challenges remain, necessitating improved stability and sensitivity, optimized detection conditions, and reduced production costs. Notwithstanding, phage display-based nano immunosensors exhibit promising potential, offering a new, safe, and swift detection technology for ensuring environmental water safety. Future research should focus on addressing these challenges, further enhancing performance, and expanding the applicability of phage display-based nano immunosensors in cholera toxin detection.

## Preparation and functionalization of nano immunosensors

**Figure 2:**
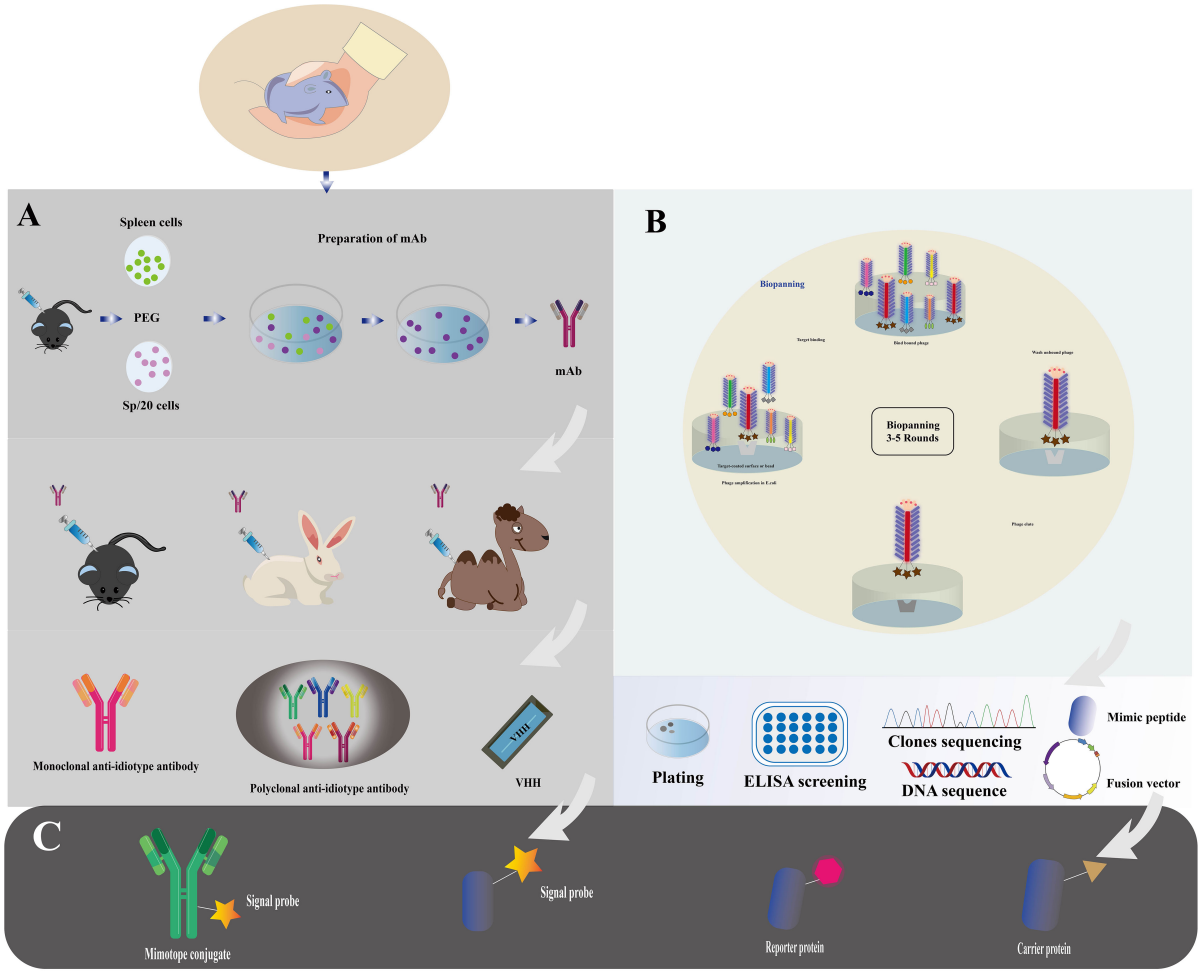
Immunosensor mimotope preparation schematic diagram. The preparation of mimotopes for use in immunosensors typically involves immunization or biopanning to obtain the mimotopes. Anti-idiotype antibodies can be prepared through immunization **(A)**, while mimotope peptides can be obtained through phage biopanning **(B)**. To expand their applications, mimotopes can be conjugated with signal probes or fused with reporter proteins and carrier proteins **(C)**. This can enhance the sensitivity and specificity of the immunosensor. The schematic diagram for the preparation of mimotopes includes the steps of selection, amplification, and preparation of single-strand DNA (Adapted from [44]).

The production and functionalization of nano immunosensors, encompassing mimotope peptides and anti-idiotype antibodies (51), assume pivotal roles in enabling effective cholera toxin detection. **Figure 2** illustrates the production and functionalization of these mimotope peptides and anti-idiotype antibodies. Utilizing phage display technology, simulated peptides are generated via biological panning, targeting primary antibodies of corresponding antigens (52). Anti-idiotype antibodies, encompassing monoclonal, polyclonal, and nano variants, can be obtained through immunizing animals with primary antibodies (53). To facilitate green immunoassays, functionalization of nano immunosensor mimotopes necessitates the use of carrier proteins or signaling probes as coated antigens or tracers (54, 55). Achieving functionalization is possible via chemical synthesis or molecular fusion techniques, with the latter showing greater potential for enhanced performance and specificity (56). Precise and efficient preparation and functionalization of nano immunosensors are paramount, ensuring their sensitivity, specificity, and accuracy in detecting cholera toxin in water sources.

### Phage display technology

**Figure 3:**
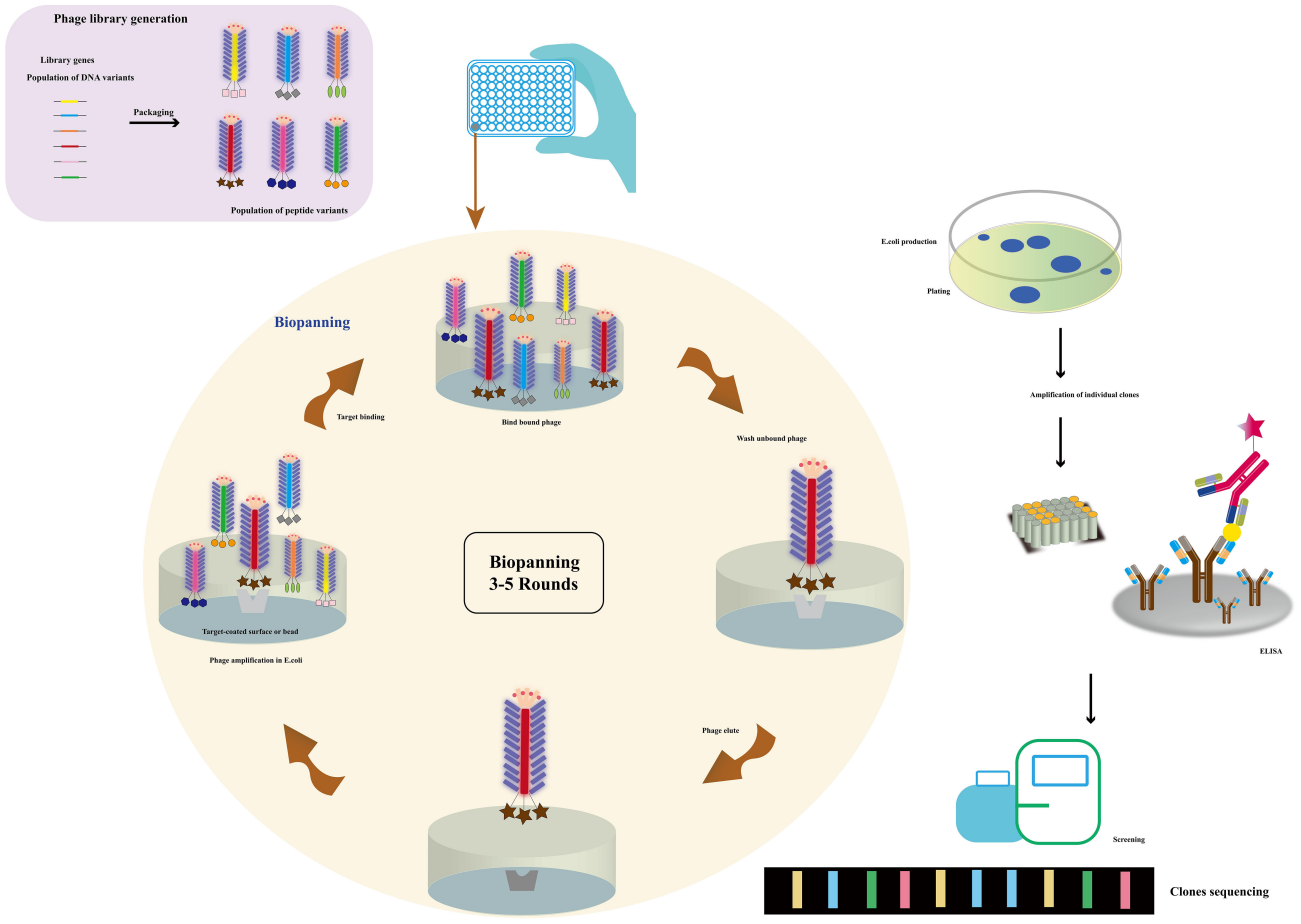
The use of a phagemid vector for phage display. Millions of library variants are cloned into a phagemid vector. By transforming E. coli with phagemids and rescuing phages, large phage libraries can be obtained. The phages with specific-binding antibodies against immobilized targets can be selected and isolated through multiple rounds of biopanning. The phages were then screened and sequenced (Adapted from [57]).

Phage display technology (**Figure 3**) emerges as the primary method for preparing mimicking peptides, vital in toxin detection. These polypeptides mimic antigen epitopes and entail genetic modification of phage DNA. By binding to a phage coat protein, peptides, proteins, and antibody fragments are expressed on the phage surface. Additionally, the introduction of exogenous DNA sequences permits the display of related genes and their products on the phage surface (58, 59). Moreover, the protein or peptide maintains its ability to recognize molecular targeted binding sites (60). In 1985, Smith pioneered the phage display technique, fusing the restriction endonuclease EcoR I with the PIII protein as a recombinant small coat protein (61). Subsequently, the shell proteins PVIII and PVI were also employed for phage display (62–64), with the innovative proposal of a dual display system (65). The M13 phages utilized in phage display can be classified into different carriers, including 3 + 3, 6 + 6, and 8 + 8 types. Phage vectors are primarily categorized into type 3/8 and type 33/88 vectors based on the number of displayed exogenous peptides (66). Phage display technology, renowned for its remarkable specificity and sensitivity, finds extensive application in nano immunosensor development for toxin detection. This versatile tool excels in epitope mapping and identifying mimotopes for diverse antigens. In essence, phage display technology stands as a potent approach for crafting highly specific and sensitive nano immunosensors, revolutionizing cholera toxin detection in water sources.

Phage display technology has revolutionized protein engineering and drug discovery. Among the most common type are random peptide libraries, showcasing millions of epitopes that aid in identifying specific binding peptides for various targets, including microorganisms, organ tissues, and nanoparticles (67). Biopanning enables the selection of peptides with high affinity and selectivity towards their targets (68). The advancement of cyclic peptides has further bolstered the technology, yielding peptides with enhanced stability and bioactivity. Notably, commercial libraries like the Ph.D-12 and Ph.D-7 phage display peptide libraries have been established, while chemical modifications can augment their abundance (69). The construction of diverse phage libraries has provided a cornerstone for biological panning of targeted binding peptides, holding tremendous potential in disease diagnosis and treatment. Additionally, chemical modification of commercial libraries facilitates the creation of phage libraries with desired abundance without genetic recombination (70). The vast repertoire of random peptide libraries, with tens of millions of epitopes, plays a vital role in disease diagnosis and treatment (71). Biopanning allows for the selection of specific binding peptides against a myriad of targets, including microorganisms like cancer cells (72–74), bacteria, viruses (75, 76), organ tissues (77), and even nanoparticles (78). Balmforth et al. (79) harnessed phage display to identify two antibody mimics, anti-cholera toxins Affimer (ACTA) -A2 and ACTA-C6, demonstrating their non-covalent binding to the unbound plane of the B subunit of cholera toxin. This discovery showcased the selective *in vivo* delivery of Affimers to motor neurons, paving the way for the development of non-viral motor neuron drug delivery vectors. Furthermore, the establishment of various phage libraries, including random peptide libraries, antibody phage libraries, cDNA phage libraries, among others, underpins the biological panning of targeted binding peptides (80).

### Structure and life cycle of phage M13

**Figure 4:**
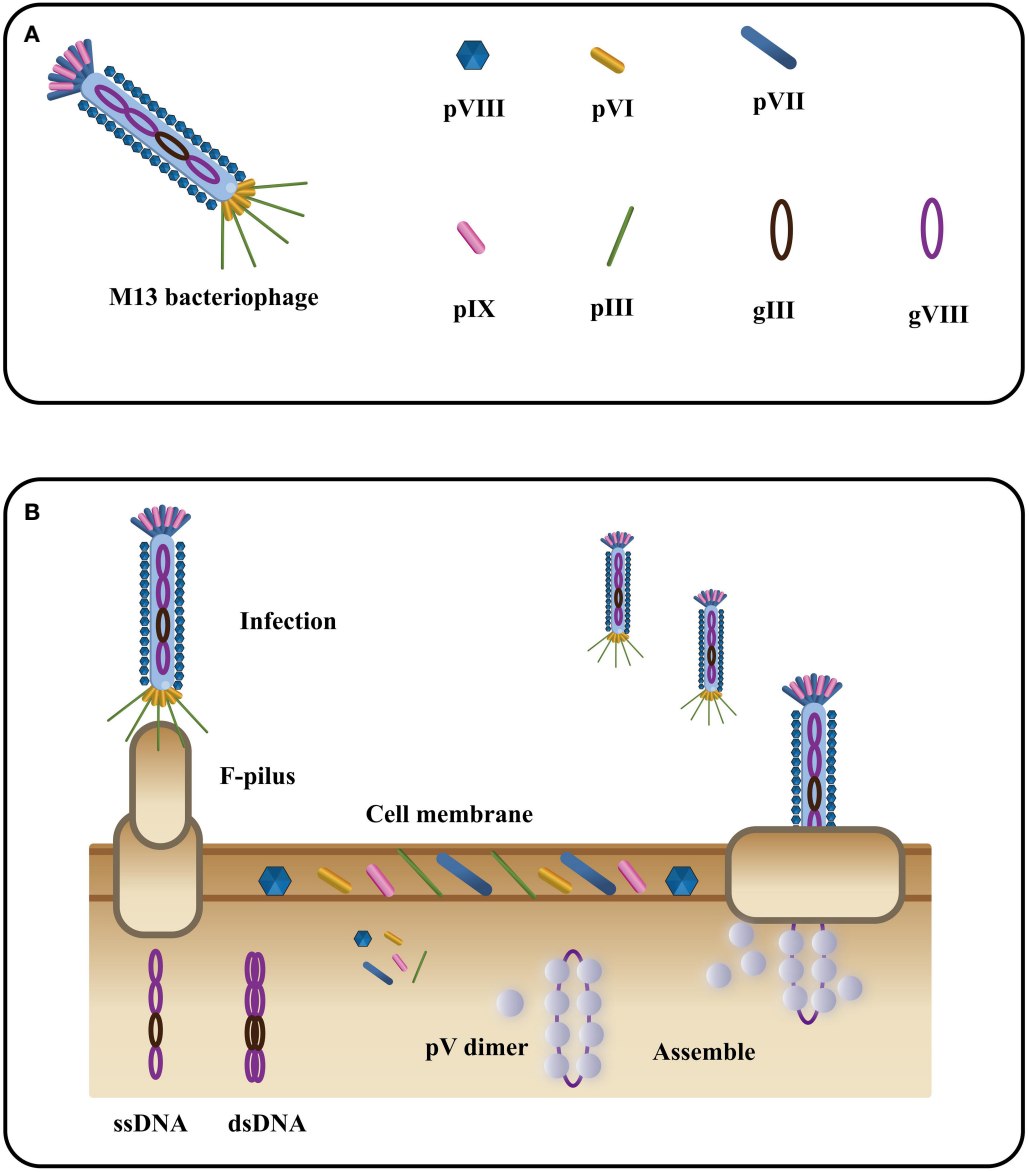
The filamentous bacteriophage’s basic structure **(A)** and life cycle **(B)** (Adapted from [83]).

Phage M13, a filamentous bacteriophage, exhibits a diameter of 6.5 nm and a length of approximately 1000 nm (81). Its structure encompasses a single-stranded DNA genome enclosed in a protein tube comprising about 2700 copies of the PVIII molecule. Additionally, four minor coat proteins, each with 5 copies, namely PIII, PVI, PVII, and PIX, are present (82) (**Figure 4A**). Diverging from other phages, filamentous phages do not cause bacterial host lysis and primarily rely on F pili to infect Escherichia coli (84, 85) (**Figure 4B**). During infection, major coat proteins detach from the phage particles and are deposited in the host, while the single-stranded DNA (ssDNA) enters the cells and undergoes conversion into a double-stranded replication form. The progeny DNA replicates via a rolling cycle mechanism and assembles into a cell core protein complex with the viral replication assembly protein gp5. With the aid of host proteins, the virions are extruded through the membrane. Subsequently, the replication assembly proteins, which cover and protect viral DNA inside the cell, are replaced by coat proteins in the cell membrane to cover and protect viral DNA outside the cell. Ultimately, the virions are extruded through the membrane with the assistance of host proteins (86). The targeted proteins play a crucial role in expressing five structural coat proteins, where each of them has one side inserted into the bacterial inner membrane, facilitating the assembly of progeny particles. Notably, on a phage particle, units comprising five proteins form a banded arrangement that encases the DNA (87). Among these coat proteins, g3p and g8p are of particular significance in the cloning and detection of recombinant phage antibodies and peptides. Typically, both g3p fusion proteins are expressed on the tip of phage M13 (88).

### Phage display to prepare simulated peptides

Phage display technology enables the cost-effective screening of novel mimicking peptides through phage peptide libraries (**Figure 3**). Using the primary antibody corresponding to the antigen as the ligand for phage display biopanning, specific phage peptides can be obtained after several cycles of affinity biopanning. These peptides simulate the amino acid epitopes and physical and chemical properties of the corresponding antigens. Through multiple rounds of biopanning, unbound phages are washed out, and the specific phage is eluted for further amplification. After 3-5 cycles of affinity biopanning, the specific phage peptides are obtained, followed by DNA sequencing and analysis for further characterization (89). The specific phages obtained through panning can simulate not only the amino acid epitopes but also the physical and chemical properties of the corresponding antigens (90). Utilizing the ph.D-12/ph.D-7 phage display peptide library kits, novel peptides that bind to the anthrax toxin receptor (ATR) with high affinity and specificity have been identified, showing potential for neutralizing anthrax toxicity in cells. Lee, Sang-Choon and colleagues (91) used phage display to select ATR-binding peptides, resulting in the discovery of two novel peptides with high affinity and specificity that could neutralize anthrax toxicity in cells. Phage display technology is widely employed for the production of recombinant antibodies (RABs), where a specific peptide is displayed on the surface of a filamentous phage particle through fusion between the gene encoding the antibody and the coat protein (pIII or pVIII). The process involves exposing the antigen-coated surface to a phage library, followed by rigorous washing to remove antibody-bound phages, which are then re-amplified by infecting E. coli. This amplification and washing cycle is repeated, with an increase in specifically bound phages expected after each round. After several rounds of washing and amplification with E. coli, antigen-specific antibody phages can be examined using various methods (92). The substitution of mimotopes for toxins offers significant advantages in enhancing laboratory and environmental safety. Phage display technology enables rapid and cost-effective amplification and purification of mimotopes, making it a practical alternative to complete antigen synthesis. Moreover, the use of self-made phage display peptide libraries provides a cost-effective approach to generate mimotopes specifically targeting the desired antigen (93, 94). This method holds immense potential for a wide range of applications, including disease diagnosis and treatment, drug discovery, and the development of toxin-free immunoassays. By leveraging phage display technology for mimotope production, researchers can explore novel avenues for safer and more efficient research and application in various scientific fields.

### Preparation of anti-idiotype antibody

**Figure 5:**
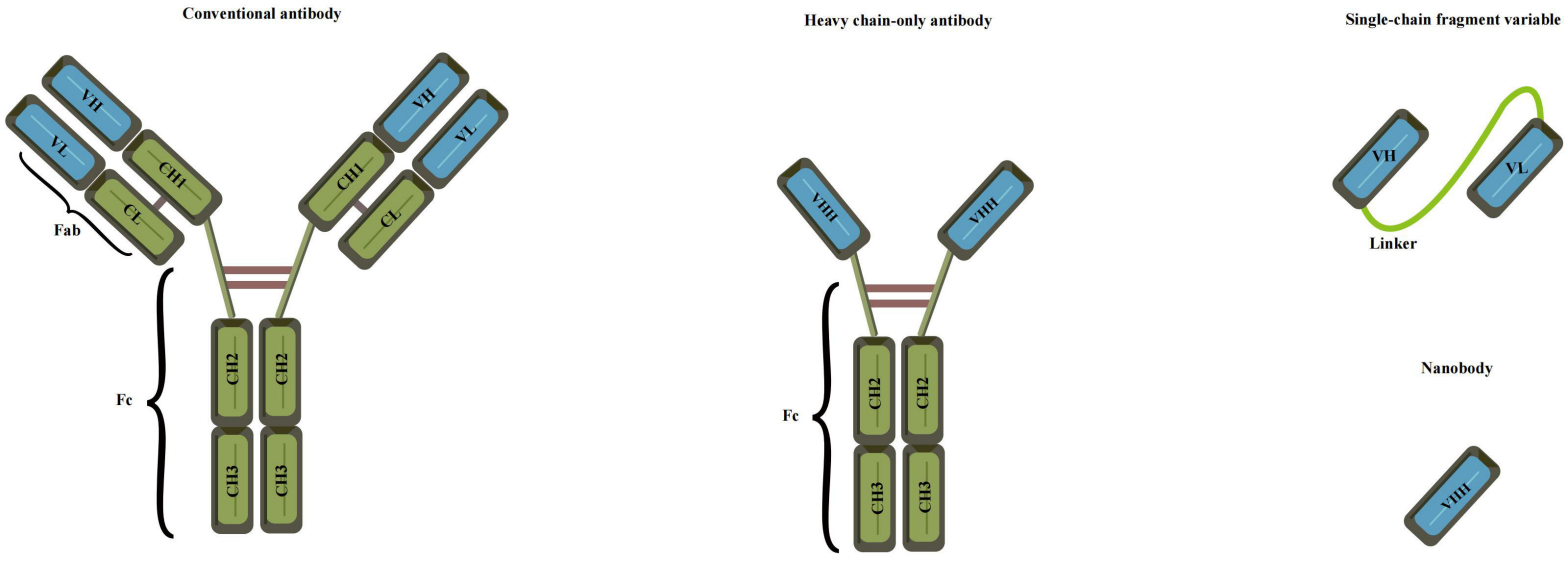
Various recombinant antibodies were compared to conventional antibodies (Adapted from [83]).

The concept of anti-idiotype antibodies, initially proposed by Jerne in 1974 as part of his “immune network theory” (95), has opened new possibilities for antigen detection. These antibodies mimic the spatial structure and biological activity of an antigen, allowing them to specifically bind to idiotypic antibodies (96). The preparation of anti-idiotype antibodies includes monoclonal, polyclonal, and nanobodies, offering a more stable conformation and enhanced environmental resistance compared to mimotope peptides. This promising attribute positions them as an attractive antigen alternative for the development of harmless immunoassays (97, 98). With their unique characteristics, anti-idiotype antibodies represent a significant advancement in immunosensor technology for safer and more reliable detection methodologies. The use of polyclonal antibodies (PABs) and monoclonal antibodies (Mabs) has been limited in simulating small haptens due to their large flat recognition surface (99). However, recent advancements have led to the emergence of nanobodies, derived from camels without light chains, offering immense potential for generating anti-idiotype antibodies. These nanobodies exhibit a smaller size, high solubility, thermostability, chemical stability, and suitability for genetic manipulation, making them a remarkable substitute in immune sensing. Notably, their variable domains can bind to antigens without domain pairing (100–103). Nanobodies, such as single-chain fragment variable antibodies (scFv) and variable domain of the heavy chain of the heavy-chain antibody (VHH), offer distinct advantages over conventional antibodies due to their smaller size (**Figure 5**). These compact nanobodies exhibit remarkable properties, including high solubility, thermostability, and chemical stability, making them exceptionally well-suited for genetic manipulation (104). As a result of these favorable characteristics, nanobodies have gained widespread recognition and have been extensively utilized in the development of toxin-free immunoassays (105). In a study conducted by Caixia Zhang et al. (106), two distinct anti-idiotypic nanobodies were isolated against OTA-specific monoclonal antibodies (mAb). Through careful examination of the primary structure, the researchers identified alterations in the complementary determining regions (CDRs). Notably, modifications were observed in CDR1, CDR2, and CDR3. The findings revealed that these anti-idiotypic nanobodies exhibited the potential to enhance the sensitivity of immunoassays. Leveraging these nanobodies as a safe substitute for conventional synthetic toxic antigens, the researchers successfully established an ELISA method that ensures both safety and efficacy.

### Functionalization of nano immunosensors

**Figure 6:**
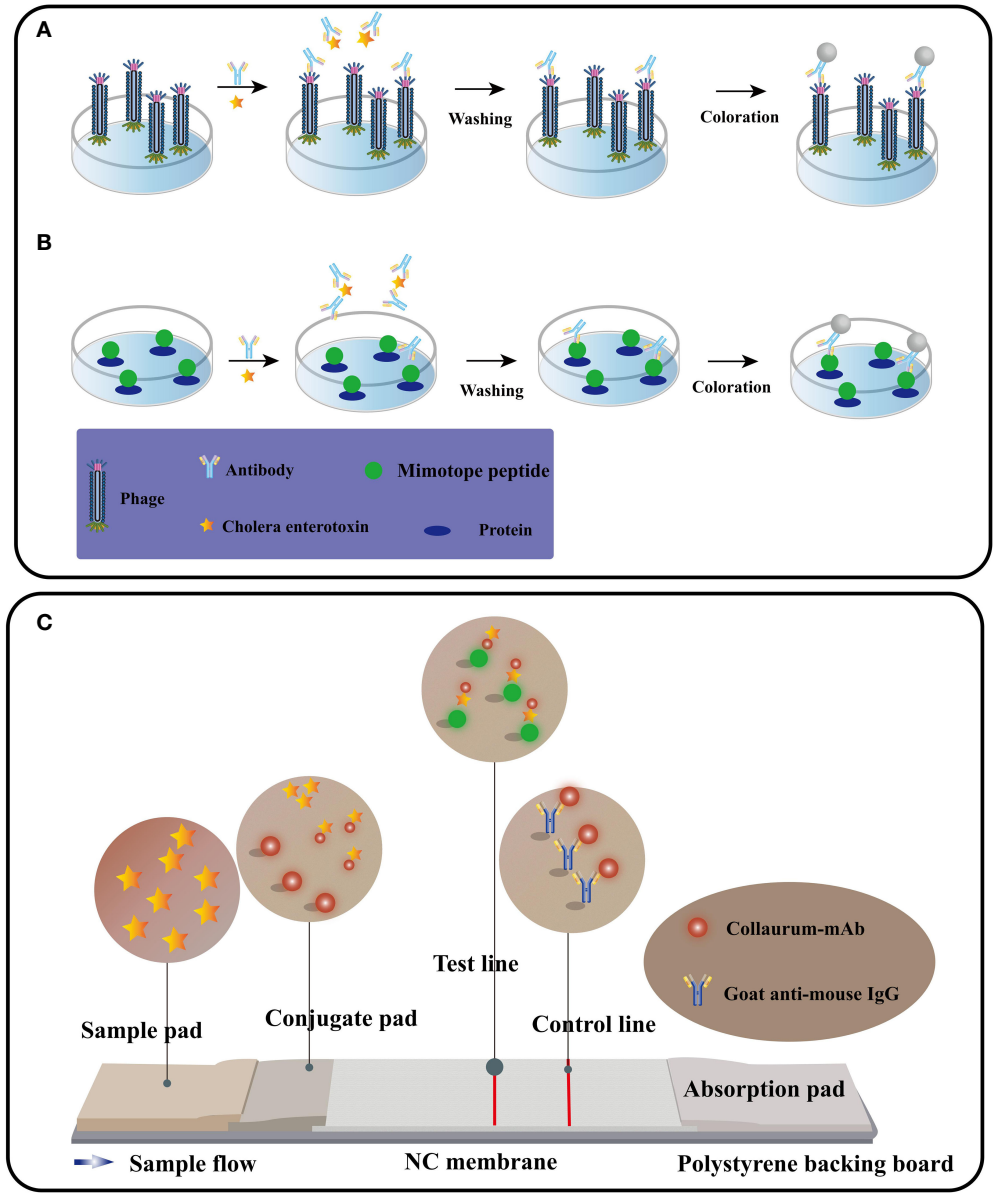
The procedure for performing competitive immunoassays using phage form **(A)** or peptide fusion protein **(B)**. Immunochromatography schematic diagram **(C)** (Adapted from [83]).

In most cases, immunosensor mimotopes are strategically labeled or fused with signal probes and reporter proteins to achieve signal amplification or serve as effective tracers (**Figure 6**). To further enhance the detection sensitivity, these immunosensor mimotopes can be labeled with biotin or composite probes. In a noteworthy study by Tong, Weipeng, et al. (107), a simulated peptide was obtained from a commercial phage display library, and to bolster sensitivity, biotin was introduced as a competitive antigen by modifying the main protein. The proposed method exhibited excellent linear detection within the range of 4.8 to 625 pg/mL, boasting an impressive detection limit (LOD) of 5.39 pg/mL. Remarkably, this LOD was approximately 26-fold lower than that of the conventional horseradish peroxidase (HRP)-based ELISA. Lu, Xin, et al. (108) have developed a remarkably sensitive electrochemical immunosensor utilizing phage display peptide derived from Bacillus thuringiensis protein. The targeted phage display peptide, obtained from the Ph.D.-12 phage display library, exhibited exceptional specificity, stability, and affinity. By integrating this selected peptide into the electrochemical immunosensor with a gold nanoparticle-modified electrode, they achieved impressive performance. The peptide-based immunosensor demonstrated a wide operating range of 0.01 to 100 ng/mL and an impressively low detection limit of 7 pg/mL. This innovative approach holds great promise for the ultra-sensitive detection of various proteins, opening up new possibilities for a range of applications. In a subsequent breakthrough, Mingyang Wang and their research team (109) introduced a screening strategy for VEGF members with a structural resemblance to VEGF165, considering its potential as a therapeutic target for various malignancies. Their aim was to significantly enhance the specificity of the selected phage monoclonal. Their efforts yielded a phage monoclonal expressing the peptide SPFLLRM, which displayed excellent affinity and specificity for VEGF165. Leveraging this specific phage-modified electrode, they successfully constructed a VEGF165 electrochemical impedance spectroscopy (EIS) immunosensor. Through meticulous optimization of experimental conditions, they achieved a linear range of 0.5-1000 pg/mL and a detection limit of 0.15 pg/mL. This phage-based EIS sensor holds promising prospects for applications in diagnostics and therapies, presenting a significant advancement in the field.

Nano immunosensors have emerged as a cutting-edge technology with tremendous potential for diverse applications, including disease diagnosis, drug discovery, and environmental monitoring. Beyond the conventional methods of labeling or fusing with signal probes and reporter proteins, researchers have explored innovative ways to further enhance the performance of these sensors by functionalizing them with various nanomaterials. Gold nanoparticles (AuNPs), for instance, have proven to be valuable in modifying the electrode surface or serving as labels for the mimotope. Such modifications lead to improved sensitivity and stability of the immunosensor. Similarly, carbon nanotubes (CNTs) have been employed to enhance the electrode surface area and facilitate electron transfer, elevating the overall performance of the nano immunosensor. Additionally, other nanomaterials, including graphene oxide (GO), quantum dots (QDs), and magnetic nanoparticles (MNPs), have also been utilized for the surface modification of electrodes or labeling of mimotopes. By doing so, researchers have achieved heightened detection sensitivity and selectivity in these advanced immunosensors. The functionalization of nano immunosensors with diverse nanomaterials showcases a promising avenue to further enhance their capabilities and widen their scope of applications.

## Application of nano immunosensors in the detection of cholera toxin

Nano immunosensors have gained widespread recognition as a valuable tool for cholera toxin detection, primarily due to their ability to obviate the need for competing antigens and tracers, streamlining the assay process and enhancing efficiency.

### Cholera toxin detection based on immunosensor mimotopes

Cholera toxin, an AB5 toxin comprising A (CTA) and B subunits (CTB) (110), shares structural similarities with other toxins, such as Escherichia coli thermal instability toxin (111) or Shiga-like toxin (112). Consequently, when developing mimotopes for cholera toxin detection, meticulous attention must be given to ensuring their specificity. This crucial aspect safeguards against potential false-positive results arising from cross-reactivity with similar toxins. Thus, stringent validation of the mimotopes is essential before practical implementation in the field (113). As delineated above, cholera toxin (CT) is a sophisticated protein complex consisting of A and B subunits. The B-subunit adeptly enters host cells by binding to ganglioside GM1, subsequently embarking on a journey to the endoplasmic reticulum. On the other hand, the A-subunit plays the role of an ADP-ribosyltransferase, catalyzing the transfer of ADP-ribose from NAD+ to specific target proteins. This enzymatic activity, orchestrated by the A1 chain of the A subunit, precipitates a cascade of events, culminating in the manifestation of watery diarrhea. Upon successful translocation of the A1 chain into the cytoplasm via the unfolded endoplasmic reticulum, a remarkable evasion from proteasomal degradation ensues. Swiftly, the A1 chain finds its equilibrium, resuming its role as a potent ADP-ribosyltransferase. Herein lies the crux of its toxicity, as the ADP ribosylation of the heterotrimeric G protein Gsa ensues. This relentless activation of adenylate cyclase fuels an unprecedented surge in intracellular levels of cyclic AMP (cAMP). Within intestinal cells, the expeditious surge in cAMP production orchestrates chloride secretion, inevitably triggering substantial water loss. The profound consequence manifests as the hallmark symptom of cholera: relentless, watery diarrhea (114).

The use of immunosensor mimotopes as recognition components has gained significant traction owing to their ability to overcome limitations while seamlessly integrating into pharmaceutical processes (115). These unique components possess the capacity to spontaneously self-assemble into specific nanostructures through non-covalent interactions, rendering them ideal molecular elements for constructing cutting-edge detection platforms (116). For instance, a remarkable breakthrough came from the research team led by Jong Min Lim (117), who harnessed the power of M13 phage display to develop an affinity peptide specifically targeting the B subunit of Vibrio cholerae. Rigorous evaluations employing ELISA confirmed the robust binding affinity, while local surface plasmon resonance (LSPR) and surface-enhanced Raman spectroscopy (SERS) were employed to meticulously assess the performance of the prepared biosensors. Impressively, the LODs achieved by LSPR and SERS were recorded as 1.89 ng/mL and 3.51 pg/mL, respectively. The sensor stands as a non-labeling, non-interference solution for the sensitive detection of cholera toxin, presenting a solid foundation for cholera monitoring and control. In a similar vein, Alejandro D. Montaner’s research team made noteworthy strides by identifying small peptides capable of binding to GM1 through the phage display of random peptides, with a focus on exploring their immunogenicity through chemical coupling (118).

### Cholera toxin detection based on phage-ELISA

An exemplar application of immunosensor mimotopes in toxin detection lies in the novel cholera toxin (CT) assay, accomplished through phage-ELISA. Traditionally, competitive antigens are indispensable for immunoassays targeting small molecules, necessitating the laborious synthesis of substantial quantities of these toxic small-molecular toxins. Leveraging mimotopes in immunoassays can circumvent the utilization of such hazardous compounds. In comparison to alternative analytical methods, immunoassays offer numerous advantages for rapid detection, boasting heightened sensitivity, augmented specificity, and simplified sample preparation (119). The inherent diminutive size of specific toxin mimotopes presents a challenge in their direct fixation to solid surfaces for immunoassays. Instead, these simulated peptides are ingeniously conjugated with specific proteins to generate fusion proteins amenable for immunoassays (120). A direct and effective approach involves utilizing M13 phages as coated antigens, in conjunction with the specific mimotope. Among the diverse immunoassay techniques, ELISA stands as a widely employed method for toxin detection. Consequently, employing specific mimotopes as antigen-coated phages within ELISA (phage-ELISA) presents a feasible and promising avenue (121). The mimotope, initially selected from a phage display library, underwent biotinylation for subsequent use as a competing antigen in the ELISA assay. To ensure specific recognition by the anti-CT monoclonal antibody, the mimotope was ingeniously fused to the p3 protein of the M13 phage. Detection of the biotinylated mimotope was achieved through the application of streptavidin-labeled polymeric horseradish peroxidase (HRP). Remarkably, this phage-ELISA approach exhibited remarkable sensitivity, boasting a detection limit of 0.05 ng/mL, surpassing traditional ELISA methods by 200-fold. By employing mimotopes in phage-ELISA assays, this method not only ensures enhanced sensitivity but also offers a safer and more efficient alternative, obviating the necessity for toxic small-molecule toxins.

Mimotopes have ushered in a revolutionary era in toxin detection, introducing a safer and more efficient alternative to conventional methods. Leveraging mimotopes within phage-ELISA assays has been scientifically demonstrated to augment sensitivity while concurrently reducing the reliance on toxic small-molecule toxins. Zhuolin Song and his accomplished team have engineered a groundbreaking ochratoxin A (OTA) assay using phage-ELISA, boasting an exceptional detection limit of 2.0 pg/mL (122). The pivotal element of this assay involves a simulated peptide meticulously screened from the M13 phage library, meticulously biotinylated, and subsequently employed as a competing antigen. Additionally, a heptapeptide simulated epitope, ingeniously fused to the p3 protein of M13, provided targeted recognition of the anti-OTA monoclonal antibody. By modifying the capsid p8 protein with biotin molecules, the team aptly loaded streptavidin-labeled polymeric horseradish peroxidase (HRP). Exhibiting remarkable sensitivity, this approach impressively outperforms traditional ELISA methods with an OTA detection limit 250 times lower. Furthermore, the integration of mimotopes has opened doors to the use of colloidal gold test strips, a rapid and cost-effective toxin detection method. Furthermore, Weihua Lai and colleagues have spearheaded the development of colloidal gold test strips tailored for OTA detection, culminating in an impressive detection limit of 10 ng/mL (123). Through meticulous panning from a random pool of seven-peptide phages, a transformative test paper was fashioned, ingeniously circumventing the problem of toxicity arising from direct toxin usage. By harnessing the potential of mimotopes within multi-immunoassay systems, efficiency, cost-effectiveness, and sensitivity in toxin detection can be markedly enhanced. Indeed, the incorporation of mimotopes in toxin detection presents a promising pathway towards elevating the safety and efficacy of toxin detection methodologies.

### Cholera toxin detection based on single-strand fragment variable antibody

In the realm of cholera toxin detection, the utilization of single-strand fragment variable (ScFv) antibodies emerges as a beacon of promise. ScFv epitomizes a recombinant protein formed by the fusion of the variable heavy chain region (VH) and variable light chain region (VL) of the antibody through a short peptide linker (124) (**Figure 5**). This strategic design imbues ScFv with a trifecta of virtues: a petite structure, enhanced permeability, and remarkable affinity. Owing to its diminutive molecular weight, formidable permeability, and exceptional affinity, scFv has garnered extensive usage in diverse domains, spanning tumor treatment (125), infectious disease prevention (126), and the detection of food safety residues (127). The advent of phage display as a remarkably efficient *in vitro* selection technique has positioned scFv as a compelling alternative to conventional antibodies. Through judicious mutation strategies, the affinity of scFv can be substantially augmented (128). This enhancement was vividly elucidated by the meticulous investigation of Lakzaei et al. (129), wherein they judiciously compared three distinct strategies: soluble antibody capture, PH step elution, and conventional panning, ultimately enriching specific antibody clones targeting diphtheria toxoid. Fifteen phage ScFV-positive clones against diphtheria toxoid were successfully isolated using the soluble antibody capture method. Conventional panning and PH step elution techniques yielded 9 and 5 positive phage ScFV clones, respectively. The soluble scFv fragments exhibited neutralizing activity ranging from 0.15 to 0.6 μg against the cytotoxic dose of diphtheria toxin, underscoring the effectiveness of soluble antibody capture in isolating specific scFv fragments. Embracing ScFv antibodies holds promise in enhancing the sensitivity and specificity of cholera toxin detection. Subsequently, Maryam Alibeiki et al. (130) employed a phage display library to select numerous antibody clones targeting clostridium perfringens toxoid ETX. The enrichment of anti-ETX-specific clones was achieved through binding to immobilized antigens, followed by elution and phage proliferation. After multiple rounds of binding selection, ELISA analysis confirmed the high affinity and specificity of the isolated clones for ETX. In a recent study, Shadman et al. (131) successfully identified novel single-chain fragment variable (scFv) antibodies against the pseudomonas aeruginosa exotoxin A domain I (ExoA-DI) from a human scFv phage library. To achieve this, the recombinant ExoA-DI of pseudomonas aeruginosa was expressed in Escherichia coli and subsequently purified using a Ni-NTA column for the screening process of the human antibody phage library.

The development of recombinant antibodies against toxins through phage display technology presents a promising avenue for diagnosing and treating infections. The screening of human antibody phage libraries allows for the discovery of antibodies from naive antibody gene banks, which in turn can be utilized to create novel therapeutic agents targeting toxin-related diseases. A new screening procedure has been devised to prevent the elimination of rare specific clones, enabling the identification of highly reactive phage clones. In the case of exotoxin A from Pseudomonas aeruginosa, the purified scFv antibodies demonstrated remarkable specificity and reactivity with both the recombinant domain I and the full-length natural exotoxin A. This exciting finding lays the foundation for the potential development of new therapeutic agents to combat pseudomonas aeruginosa infections. Altogether, phage display technology represents a powerful tool for the development of recombinant antibodies against toxins, revolutionizing the diagnosis and treatment of infectious diseases (132, 133).

### Cholera toxin detection based on immunosensor anti-idiotype antibody

**Figure 7:**
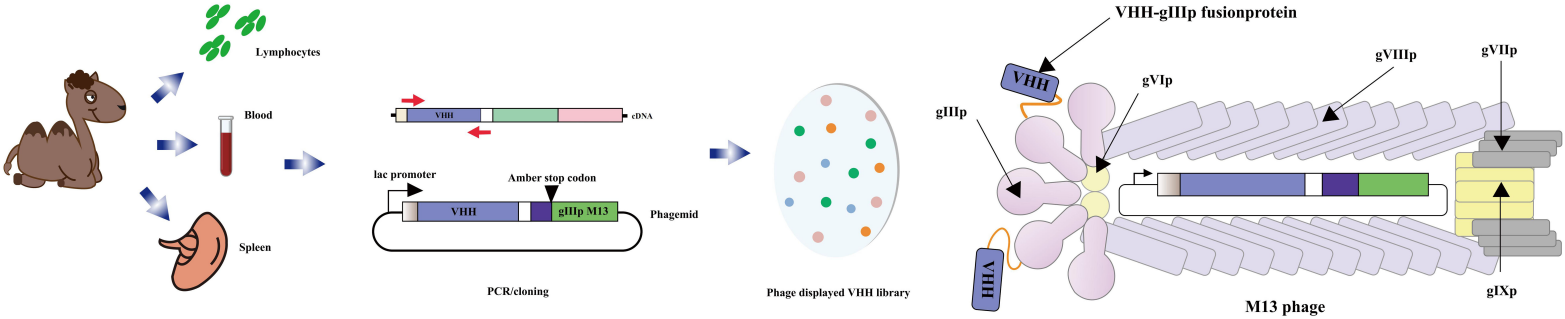
Schematic diagram of the M13 phage vector used for clone library of VHH. The open reading frame of the V-domains from the heavy chain-only antibody was PCR amplification from lymphocytes in immunized animals. The PCR product was cloned into a phage vector downstream of the promoter, in the box upstream of epitope tags, and followed by the coding sequence for the M13 phage head capsid protein gIIIp. The phage was then transfected into Escherichia coli that could be read by codon. After infecting bacteria with helper phage, a library of recombinant phage particles was obtained from the supernatant (Adapted from [139]).

The anti-idiotype antibody, located in the variable region of the immunoglobulin with specific antigenic determinants, serves as a secondary antibody targeting a particular type of primary antibody (134, 135). Numerous anti-idiotypic antibodies have been developed, effectively targeting both large and small molecules, and have found applications in diagnosis and immunoassays (136–138). A captivating discovery was made in 1993 when camelidae antibodies were found to possess no light chains, comprising solely heavy chain antibodies. These unique antibodies feature a single variable domain (VHH) forming the variable domain (VH), giving rise to what is known as a nanobody. Notably, camel immune VHH libraries can be cloned using the M13 phage display vector. The M13 phage display vector, commonly employed for cloning such libraries, facilitates the generation of heavy chain-only antibodies lacking light chains. The V-domains from these antibodies are amplified from cDNA derived from lymphocytes found in peripheral blood, lymph nodes, or spleen of an immunized animal, subsequently cloned into the phage vector. Following transfection into Escherichia coli and infection with a helper phage, a library of recombinant phage particles can be harvested from the supernatant (**Figure 7**). Given their small size and ease of manipulation, anti-specific nanobodies have emerged as valuable tools for diagnostic and therapeutic purposes (140–143). Moreover, anti-idiotypic antibodies have found utility beyond their traditional applications, extending to immunoassays targeting small molecules. An illustrative example is the anti-idiotypic nanobody-phage display-mediated immuno-polymerase chain reaction method, enabling simultaneous quantitative detection of multiple toxins. In a recent investigation conducted by Dong, Sa et al. (144), anti-Cry1A polyclonal antibodies were employed as antigens to screen for anti-variant antibodies capable of mimicking the Cry1A toxin, using a phage display human domain antibody library. Following four rounds of biopanning, five positive clones exhibiting binding activity were identified, with clone D6 displaying a remarkable inhibitory effect on the binding of Cry1A toxin and anti-Cry1A polyclonal antibodies. D6 was characterized as a subtype anti-idiotypic antibody, proficient in mimicking Cry1A toxin and competitively binding to anti-Cry1A polyclonal antibodies. Intriguingly, bioassay results demonstrated that D6 possessed discernible insecticidal activity. The study lays a foundation for the development of toxin simulators, offering promising applications in the domains of agriculture and environmental protection.

**Figure 8:**
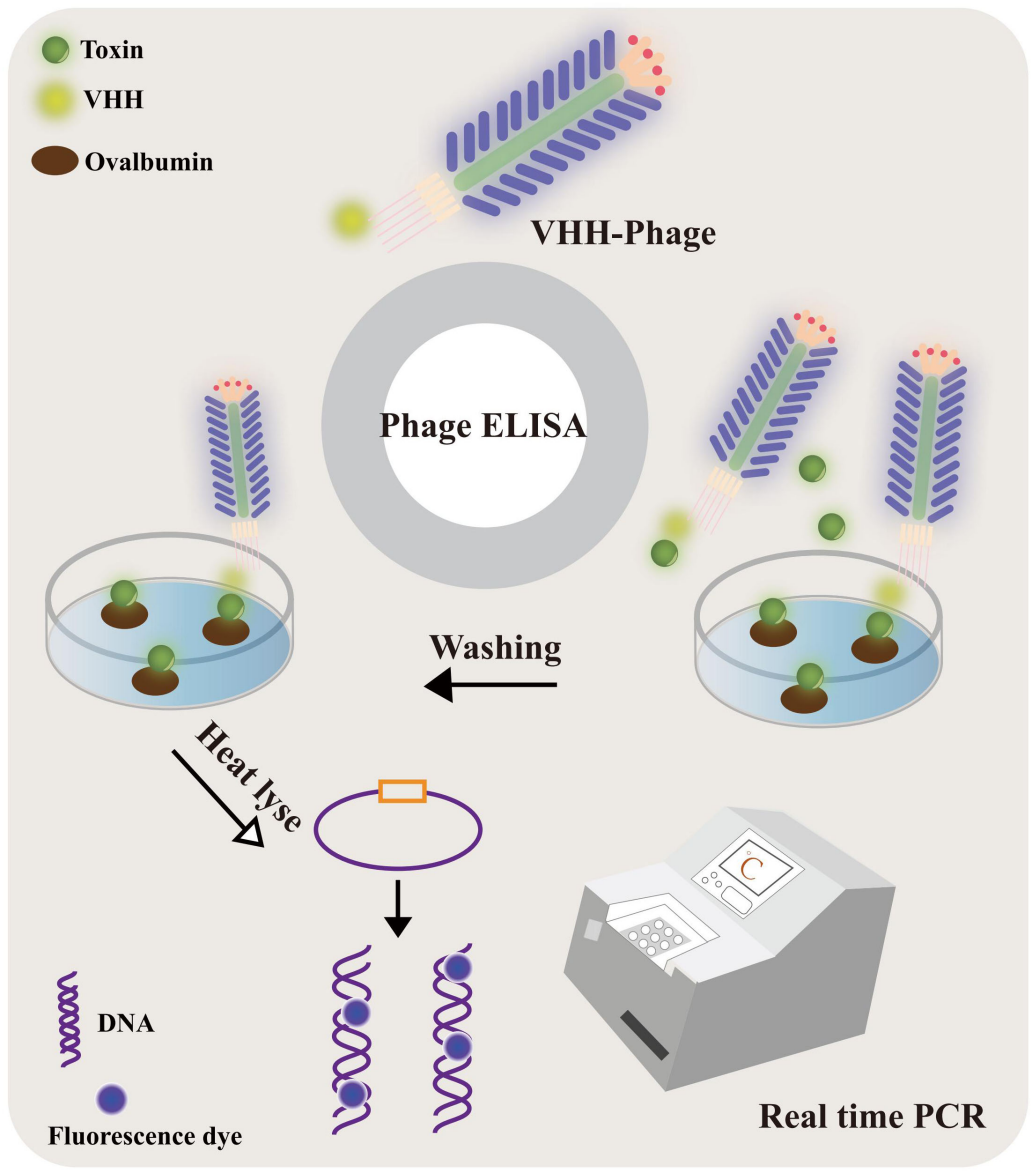
The VHH-phage-based PD-IPCR process. VHH-phages and toxin were mixed and incubated with ovalbumin-conjugated toxin precoated on a solid surface. The VHH-phage with toxin was washed out and the excess VHH-phage was fixed. The VHH-phage DNA was released by heat lyse as a template for real-time PCR.

Notably, Xu, Chongxin, et al. (145) have achieved a significant breakthrough in the field of toxin monitoring by developing a highly sensitive anti-microcystin single-chain variable fragment (scFv). The application of this scFv in an established ELISA showcased promising prospects for ultra-sensitive detection in water samples. Besides competitive immunoassays, innovative nanobody-based methods have also emerged, among which the phage display mediated immunopolymerase chain reaction (PD-IPCR) technique deserves special mention. PD-IPCR harnesses recombinant phage particles as readily available reagents for IPCR, obviating the need for conventional monoclonal antibodies (146), as shown in **Figure 8**. This technique exhibits tremendous potential as an ultra-sensitive assay for small molecules, rendering it a valuable tool for monitoring toxins in both environmental and clinical samples. Furthermore, Rezaei, Zahra S. et al. (147) have made strides in the detection of vascular endothelial growth factor (VEGF) by developing a precise PCR-conjugated phage display system using VHH nanobodies. This innovative approach, known as PD-IPCR, presents new avenues for VEGF detection, highlighting the versatility and potential of nanobody-based methods in diagnostics and therapeutics. In a remarkable development, a novel system has been devised utilizing anti-VEGF monoclonal antibodies as specific binding agents within a sandwich immunosorbent assay platform. Strikingly, the same anti-VEGF phage particles that serve as anti-VEGF reagents in the PD-IPCR method also function as DNA templates simultaneously. During validation, the anti-VEGF phage ELISA exhibited an impressive linear range of 3-250 ng/ml, setting a new standard with a remarkable detection limit (LOD) of 1.1 ng/ml. By leveraging the PD-IPCR method, the linear range for VEGF expanded to an unprecedented 0.06-700 ng/ml, with an astonishingly low detection limit of 3 pg/ml. Such an advanced method displayed commendable sensitivity, yielding a serum recovery rate of 83-99% and a relative standard deviation of 1.2-4.9%, thus rendering it highly suitable for clinical analysis. The method’s practicality and efficacy were evident as it was successfully applied to the clinical determination of VEGF in human serum samples. Impressively, the results obtained showed a strong correlation with conventional ELISA findings. These significant findings underscore the immense potential of the PD-IPCR method, positioning it as a valuable tool for clinical diagnosis and other multifaceted applications in the realm of VEGF detection.

A groundbreaking study by Ren et al. has unveiled an innovative approach utilizing anti-idiotype nanobody-phage display mediated immunopolymerase chain reaction for the simultaneous and quantitative detection of total aflatoxin and zearalenone in cereals (148). Impressively, this method exhibited exceptional sensitivity and specificity, boasting a linear range of 0.01-100 ng/mL for zearalenone (ZEN). Similarly, Xianxian Wang et al. have made substantial strides by developing a highly sensitive and specific immunoassay for the quantitative determination of ZEN, employing the anti-idiotype variable domain of heavy-chain antibodies (VHH) PD-IPCR (149). Through four cycles of refinement, a remarkable anti-idiotype VHH phage clone was isolated, and the LOD of PD-IPCR based on the VHH phage stood at an astonishing 6.5 pg/mL, surpassing phage ELISA by approximately 12-fold. The promise of high throughput analysis in practical applications became evident with the successful application of this method in ZEN detection of grain samples using liquid chromatography-tandem mass spectrometry (LC-MS/MS). These groundbreaking studies collectively underscore the immense potential of anti-idiotype PD-IPCR methods, offering a gateway to ultra-sensitive mycotoxin detection in cereals. The utilization of PD-IPCR not only enhances sensitivity and expands the linear range compared to phage ELISA but has also been verified for ZEN detection in grain samples through LC-MS/MS validation. In addition to PCR-based analysis, loop-mediated isothermal amplification (LAMP) represents another cutting-edge technique for the rapid and simple detection of target nucleic acids (150). The impact of this groundbreaking method is evident in a myriad of diagnostic fields, including bacterial, viral, and parasitic pathogen detection (151, 152), as well as disease diagnosis (153). Among the innovative adaptations of the loop-mediated isothermal amplification technique, the immunolamp (iLAMP) assay has emerged as a particularly promising tool for toxin detection. Renowned for its specificity, visual identification capabilities, and isothermal amplification, the iLAMP assay follows a well-defined procedure. It involves applying antitoxin monoclonal antibodies to the base of a glutaraldehyde-treated PCR tube, followed by the addition of the sample extract and anti-idiotype nanobody phage to the tube. Following the reaction, unbound phage is meticulously removed, and the LAMP solution is introduced to the test tube for the amplification process. With encouraging results in toxin detection, the iLAMP assay holds significant potential as a rapid and straightforward detection tool across diverse fields of application.

**Figure 9:**
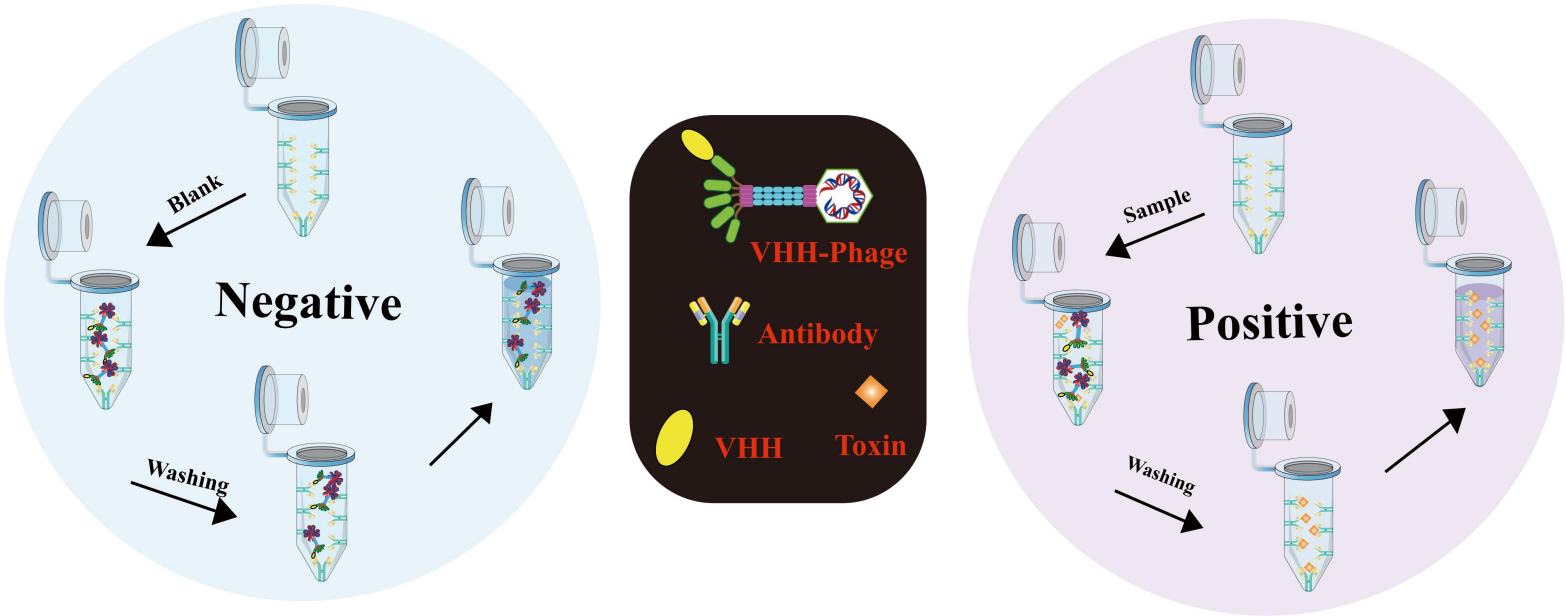
Detailed procedure for iLAMP detection (Adapted from [155]).

In a groundbreaking study led by Chen Dailing et al. (154), the rapid detection of virulence-related genes in Vibrio cholerae (ace, zot, cri, and nanH) was achieved using the highly efficient Loop-mediated Isothermal Amplification (LAMP) visualization method. To accomplish this, the researchers designed and synthesized three pairs of molecular probes for each gene. Positive results were readily identified by the development of a vibrant green color under visible light or green fluorescence under ultraviolet light (302nm). Remarkably, the LAMP method exhibited detection limits ranging from 1.85 to 2.06 pg per genomic DNA response, surpassing the sensitivity of standard PCR. These results highlight the potential of LAMP as an effective and sensitive tool for the rapid detection of Vibrio cholerae and its associated virulence factors. Another notable adaptation of LAMP, the immunolamp (iLAMP) assay, has emerged as a powerful approach for toxin detection, owing to its specificity, visual identification capability, and isothermal amplification. The fundamental procedure involves coating the bottom of a glutaraldehyde-treated PCR tube with antitoxin monoclonal antibodies (mAb), followed by the addition of sample extract and anti-idiotype nanobody phage to the test tube. These nanobody phages effectively mimic antigens (toxins) in the immune response, resulting in competition between phages and toxins for binding to mAb. After the reaction, unbound phages are meticulously removed through a washing step, and the LAMP solution is introduced to the test tube for the subsequent amplification process. As a consequence, if the sample contains toxins, the mixture in the tube retains a distinctive purple color (indicating a positive result); otherwise, it turns sky blue (indicating a negative result) (155) (**Figure 9**). This approach holds remarkable potential for the rapid and straightforward detection of toxins, making it a valuable tool in various fields, including food safety and environmental monitoring.

## Simultaneous determination of multiple cholera toxins

Currently, a diverse array of technologies is available for the detection of biotoxins, encompassing chromatography, immunochemical determination, and electrochemical methods (156–158). Notably, multiple immunochromatography (mICA) strips have been devised to enable simultaneous monitoring of multiple toxins (159). These innovative strips utilize various small molecular toxins or antibody-conjugated fluorescent nanoparticles, such as colloidal gold particles (160), fluorescent microbeads (161), and carbon nanoparticles (162). In light of the importance of efficient surveillance of freshwater sources, the electrospray ionization (ESI) liquid chromatography-mass spectrometry (LC-MS) technique has emerged as a formidable tool for biotoxin detection. In parallel, the development of a multiplex lateral flow immunoassay (LFA) has proved instrumental in the detection of primary marine biotoxin groups, including amnesic shellfish poisoning. Lastly, notable strides have been made in the development of multiple immunoassays targeting diverse biotoxins through the implementation of immunosensor mimotopes. This approach holds tremendous promise for the creation of more efficient and accurate detection methods specifically tailored for cholera toxin detection. These advancements signify significant progress in the field of biotoxin detection, paving the way for enhanced diagnostic capabilities and improved safety measures.

### Immunoassay flow chromatography based on immunosensors

**Figure 10:**
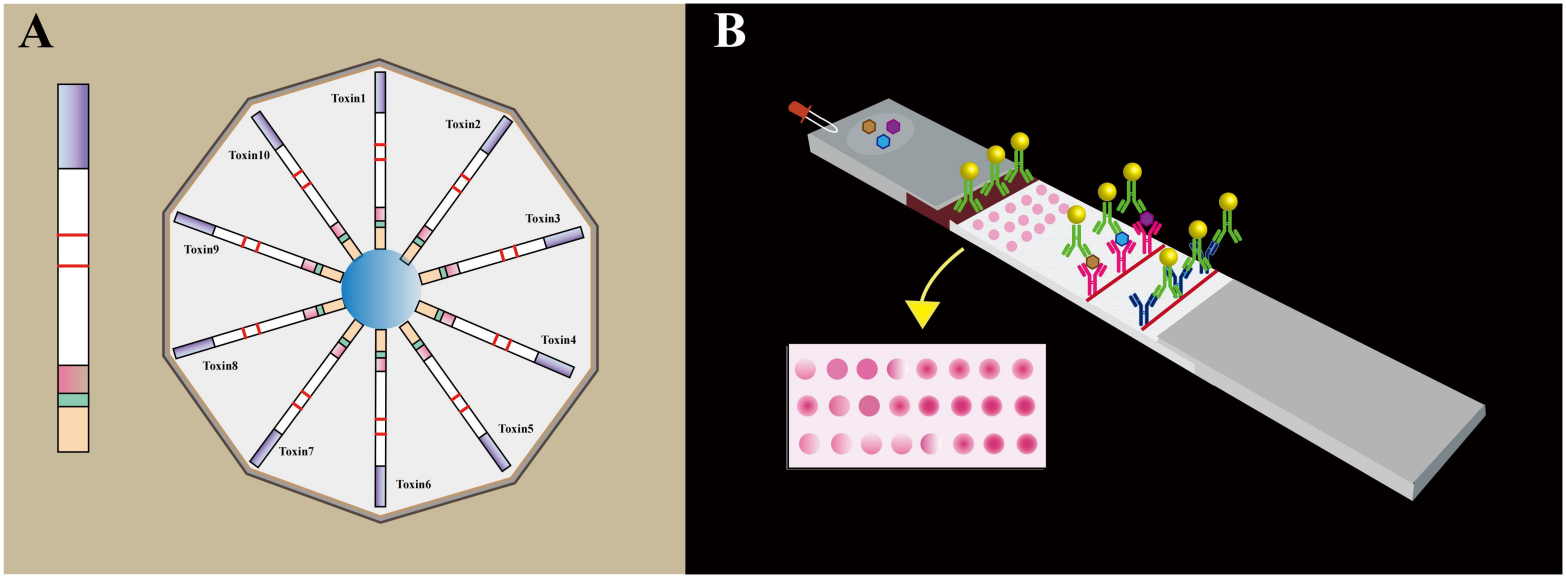
**(A)** The individual test strip for a single toxin by means of an antibody linked to a phosphorous probe. The 10 channels phosphorous probe-based lateral flow disc holds 10 detection channels (Toxin1 to Toxin10), each consisting of a single strip for the target toxin. **(B)** The schematic representation of the lateral flow microarray strip. Each of the 32 dots represents a distinct capturing agent (Adapted from [165]).

An immunoassay flow chromatography utilizing immunosensor mimotopes has emerged as a powerful method for the concurrent detection of multiple cholera toxins. Among the various screening platforms, immunochromatography (ICA) stands out as the most widely employed and well-established technique, offering advantages of simplicity, rapidity, stability, high throughput, ease of use, and cost-effectiveness (163) (Figure. 6C). Central to ICA’s functionality is the liquid test sample’s flow, which interacts with the analyte through a strip containing antibodies, leading to the accumulation of a chromogenic substance that generates a readable signal (164). Presently, researchers are actively working on developing both single and multiple assays with conventional antibodies using ICA (**Figure 10**). The integration of immunosensor mimotopes in this approach holds tremendous promise to enhance the accuracy and sensitivity of the ICA method, positioning it as an invaluable tool for the detection of biotoxins across diverse fields, such as food safety and environmental monitoring. A series of recent studies conducted by Tong and his esteemed research team have been devoted to the development of innovative immunochromatography sensors for detecting zearalenone (ZEN), exhibiting excellent reproducibility and employing a bio-safe approach (166–168). Additionally, Yan, Jia-Xiang, and colleagues have made significant strides in creating a cost-effective and highly sensitive multiplex immunochromatographic assay (mICA) that allows for the rapid detection of fumonisins B-1 (FB1), zearalenone (ZEN), and ochratoxin A (OTA) (169). This groundbreaking mICA harnesses mimotopes of FB1, ZEN, and OTA from phage display technology, skillfully integrated with maltose binding protein (MBP) as simulated coated antigens applied to the mICA test line. Impressively, the visual detection limits achieved by this novel method are an astonishing 0.25 ng/mL for FB1, 3.0 ng/mL for ZEN, and 0.5 ng/mL for OTA, all within an impressive timeframe of merely 10 minutes. Subsequent testing using real-life samples has affirmed the method’s accuracy, reproducibility, and practicality. Notably, the proposed mICA technique has been demonstrated to yield results on par with those obtained from the widely regarded ultra-high-performance liquid chromatography combined with tandem mass spectrometry (UPLC-MS/MS) in detecting FB1, ZEN, and OTA using natural samples.

### Multiple cholera toxins detection based on PD-IPCR

As discussed earlier, PD-IPCR represents an ultra-sensitive immunoassay for small-molecule biotoxin detection. Its remarkable sensitivity and specificity make it a highly promising solution for toxin detection across various domains, including food safety, environmental monitoring, and public health. Ren et al. (170) devised an innovative detection platform that integrates PD-IPCR and RT-PCR, enabling simultaneous detection of multiple toxins in stored corn. PD-IPCR, an ultra-sensitive immunoassay for small-molecule biotoxins, was combined with real-time PCR assays capable of detecting various genes, including the cholera toxin gene. This platform facilitated the concurrent detection of multiple toxins, while establishing a quantitative standard curve for comprehensive analysis. The detection limits were 0.02 ng/mL for aflatoxins and 8×10^2^ spores/g for Aspergillus section Flavi, respectively. This method holds promise for simultaneous detection of small molecular pollutants and microorganisms, introducing novel perspectives in diagnostic and public health applications. The development of this detection platform has significant potential in simultaneously detecting small molecular pollutants and microorganisms, offering fresh insights for detection technology advancements. The capability to detect multiple toxins in one sample enhances detection efficiency, accuracy, and cost-effectiveness. This has profound implications in food safety, environmental monitoring, and public health. Continued progress in biosensors, chemosensors, and assays opens doors to innovative methods for screening and identifying other biotoxins.

## Summary

Immunoassays play a crucial role in rapidly screening biotoxin residues in the environment for human health protection. However, their traditional approach involving large quantities of toxic biotoxins or complex chemical synthesis of coated antigens and tracers presents challenges. The chemical synthesis process suffers from drawbacks like intricate procedures, batch errors, low binding efficiency, and the use of organic solvents. As an innovative substitute, immunosensor mimotopes, such as simulated peptides and anti-idiotype antibodies, offer a safer option for these immunoassays. Utilizing phage display or monoclonal/polyclonal antibody technology, immunosensor mimotopes serve as competing antigens, tracers, or standard substitutes. They eliminate the need for toxic compounds and provide an eco-friendly analytical tool for biotoxin detection, addressing issues of batch errors, low binding efficiency, and organic solvent usage. Immunosensor mimotopes hold immense promise as reagents in the detection of cholera toxins.

Phage M13, widely used in mimotope biopanning, offers a versatile approach for detecting small analytes, including biotoxins, via M13 phage display technology. Construction of random peptide or recombinant antibody libraries facilitates the selection of alternative antigens or antibodies. The choice between libraries should consider specific application requirements, as each possesses distinct advantages and limitations. Random peptide libraries, simpler to construct, exhibit a broad epitope recognition range, including continuous and discontinuous sequences, allowing specific binding to various proteins or ligands. Conversely, recombinant antibody libraries offer higher affinity and specificity for the target analyte. In cholera toxin detection, the use of phage M13 in mimotope screening presents a powerful tool for alternative antigen or antibody development. Random peptide libraries demonstrate particular utility in detecting small antigens without bias. However, they may exhibit lower affinity, limiting certain applications. In such cases, recombinant antibody libraries become a preferred choice, yielding antibodies with superior affinity. Thus, selection between random peptide and recombinant antibody libraries hinges on the specific needs of the intended application, ensuring optimal results for cholera toxin detection.

In practical applications, the detection of cholera toxins often involves using fusion proteins formed by coupling random peptides with other proteins, rather than single peptides or phages alone. Multifunctional phage display technology has enabled the development of versatile biosensors based on M13 phage for detection and analysis. Through surface modifications, M13 phage gains new characteristics, such as targeted binding ability, optical properties of quantum dots, and the accumulation of magnetic nanoparticles (171). These modifications make M13 phage an ideal platform for highly sensitive and selective biosensor development in cholera toxins detection, offering improved accuracy and efficiency. In contrast to random peptides, recombinant antibodies possess the advantage of direct fixation on both solid surfaces and antigens. Isolated from libraries, recombinant antibodies like scFvs and anti-idiotype antibodies find applications in sensitive diagnosis techniques such as ELISA, PD-IPCR, and iLAMP. Additionally, recombinant antibodies expand the scope of immunoassay agents, allowing for the detection of diverse biotoxins within the same group. The use of recombinant antibodies has led to simultaneous biotoxin detection based on phage display, showcasing their advantages, including enhanced specificity, increased sensitivity, shorter detection time, and improved safety. Recombinant antibodies represent a potent tool for the sensitive and specific detection of cholera toxins. In the quest for cholera toxin screening, researchers harnessed diverse resources, including antigens for antibody preparation, proteins, and antibody samples for immunomolecular sieving. Antigens, derived from animals, triggered immune responses, resulting in specific antibodies. To enhance affinity, an animal immune repository was utilized, spotlighting antibodies finely tuned to the toxin. Nanobodies, from animals like camels, featured compact size, stability, and genetic manipulability, becoming potent alternatives to conventional antibodies. Nanobodies played crucial roles, particularly in targeted antibody selection, mimicking toxin epitopes for precise immune analyses, thus serving as high-affinity substitutes. This integrated approach supported effective cholera toxin detection and analysis (172).

Despite the significant benefits offered by Immunosensor mimotopes in cholera toxins immunoassays, certain limitations persist. The preparation of mimicking peptides and anti-idiotype antibodies can be challenging, and the selection of mimotopes from phage display peptide libraries may encounter failure probabilities. Likewise, obtaining anti-idiotype antibodies from immune animals can also pose challenges. Overcoming these limitations requires expanding the diversity of peptide libraries and enhancing screening techniques for positive clones. Ongoing research to optimize conditions for mimotope selection and screening holds the potential to further improve their utility in cholera toxins detection. Despite the challenges, mimotopes present a promising avenue for the development of safe and effective immunoassays. While immunosensor mimotopes have shown promise in cholera toxins immunoassays, some reported mimotopes have demonstrated only marginal improvements in assay performance. The use of phage display peptides can lead to complex procedures, and laboratory-synthesized mimotopes may lose binding activity in certain cases. To address these issues, further investigation into the structure of mimotopes and their interaction with receptors is essential. Site mutagenesis could be introduced to enhance the characteristics of mimotopes and optimize immunoassay performance.

This review delves into a pioneering avenue in the realm of cholera toxin detection: phage display-based nano immunosensors. Engineered bacteriophages, uniquely tailored with specific antibody fragments or mimotopes, exhibit an exceptional affinity for cholera toxin. This dynamic interplay orchestrates the generation of quantifiable signals, enabling remarkably sensitive detection and precise quantification. In the current landscape of cholera screening and detection, animals and animal-derived products play a significant role. Monoclonal antibody (mAb) based rapid diagnostic tests (RDTs) are employed, although their sensitivity is hindered by the presence of the common virulent bacteriophage ICP1 (172). A study explores phage-displayed mimotopes for a cholera vaccine, overcoming challenges posed by the toxic nature of lipopolysaccharide (LPS) (173). In contrast, phage display-based nano immunosensors emerge as an ethical and effective alternative (174). This innovative method demonstrates heightened affinity for cholera toxin, potentially transforming detection approaches, and reflecting the evolving landscape of efficient methodologies. This paradigm shift not only showcases the potential to revolutionize cholera toxin detection but also underscores its promise in advancing precision and efficacy in analytical methodologies (113, 173, 174).

## Author contributions

YL: Conceptualization, Methodology. K-DY: Writing-Original Draft, Investigation. J-FY: Resources, Methodology.D-CK: Writing Review and Editing. All authors contributed to the article and approved the submitted version.
